# Metrics of Mobility by Sex are Associated with HIV Incidence in Rural Kenya and Uganda

**DOI:** 10.1007/s10461-025-04743-6

**Published:** 2025-05-06

**Authors:** Carol S. Camlin, Sarah A. Gutin, Edwin D. Charlebois, Torsten B. Neilands, Laura B. Balzer, Maya L. Petersen, Gabriel Chamie, Craig R. Cohen, Elizabeth A. Bukusi, Moses R. Kamya, Diane V. Havlir, James Ayieko

**Affiliations:** 1 Department of Obstetrics, Gynecology & Reproductive Sciences, University of California, San Francisco, San Francisco, CA USA; 2 Division of Prevention Science, Department of Medicine, School of Medicine, University of California, San Francisco, San Francisco, CA USA; 3 Department of Community Health Systems, School of Nursing, University of California, San Francisco, San Francisco, CA USA; 4 School of Public Health & College of Computing Data Science and Society, University of California, Berkeley, Berkeley, CA USA; 5 Division of HIV, Infectious Diseases and Global Medicine, Department of Medicine, University of California, San Francisco, San Francisco, CA USA; 6 Kenya Medical Research Institute, Nairobi, Kenya; 7 Infectious Diseases Research Collaboration, Kampala, Uganda; 8 Department of Medicine, Makerere University, Kampala, Uganda

**Keywords:** Mobility, Migration, HIV incidence, Sub-Saharan Africa, Gender

## Abstract

The complex and dynamic nature of human mobility requires use of multiple measures and sex-stratified analyses to fully understand its influence on HIV acquisition risk in specific populations and settings. Longitudinal population-based studies designed to measure the influence of mobility (which includes both short and long-term, temporary and permanent changes of residence across defined geopolitical boundaries) on HIV acquisition risk are rare, but needed to understand the ongoing challenges that mobility poses to progress in ending HIV. Incidence of HIV acquisition over 3 years was measured in mobile and non-mobile adults in 32 rural communities in three regions of Uganda and Kenya participating in the Sustainable East Africa Research in Community Health (SEARCH) trial (NCT01864603) from 2013–2017. Poisson regression models were used to estimate incidence rate ratios (IRRs) for HIV acquisition among categories of mobile relative to non-mobile adults, with sex-stratified multivariable models adjusted for community clustering using robust estimators. At baseline, 11,337 adult residents (9.7%) reported living at least 1 month outside their community in the past 12 months (8% women, 11.8% men, *p* < 0.001). By year three, 8502 HIV incidence cohort members (7.3%) had out-migrated (7.7% women, 7.1% men, *p* = 0.046), and 5747 adults remaining in the cohort (4.9%) reported living at least 1 month outside of their community in past 12 months (4.1% women, 5.9% men, *p* < 0.001). Over 3 years, the risk of HIV acquisition was 1.9 times higher in those living ≥ 1 month outside their community in the past 12 months compared to those who had not (Adj IRR = 1.88, 95% CI 1.48–2.38). HIV acquisition risk was 42% higher among men who spent some nights away in the past month at baseline (Adj IRR = 1.42, 95% CI 1.11–1.81), but not for women (Adj IRR = 1.06, 95% CI 0.85–1.32). Mobility was significantly associated with a risk of HIV acquisition among adults in rural communities in Kenya and Uganda in 2013–2017. The effect of mobility on HIV incidence varied by both sex and pattern (frequency, duration) of mobility (SEARCH ClinicalTrials.gov number, NCT01864603).

**Supplementary Information:**

The online version contains supplementary material available at 10.1007/s10461-025-04743-6.

## Introduction

The HIV/AIDS epidemic is declining in sub-Saharan Africa, yet progress towards reductions in HIV incidence has slowed in recent years; in 2022, UNAIDS reported the smallest annual decline in new HIV infections (3.6%) since 2016 [1]. Human mobility, which includes both short and long-term, temporary and permanent changes of residence across defined geopolitical boundaries (such as a nation, state, province, or county), poses a challenge to efforts to end HIV/AIDS [2–8]. Highly mobile adults, along with youth and men in specific settings, face barriers to engagement in otherwise highly successful national HIV prevention and treatment programs and large-scale intervention studies [6–16], hampering efforts to meet the 2030 global targets.

About 50% of new HIV infections globally in 2022 occurred in sub-Saharan Africa [17, 18], and the epidemic continues to be concentrated in Eastern and Southern Africa, where approximately 500,000 individuals newly acquired HIV in 2022 [1–63]**.** Treatment coverage in sub-Saharan Africa also remains incomplete: there were 20.8 million [17.4–24.5 million] persons living with HIV (PLHIV) in the region in 2022, of whom 17.3 million were accessing antiretroviral therapy (ART) [17]. Human mobility is also highly prevalent in the region, with the world’s highest levels of intracontinental migration [19–21]. Mobility is a key element of livelihoods in the region, where individuals not only migrate to urban areas, but also from rural to other rural or peri-urban settings for work [22, 23]. Individuals also periodically circulate to other places where members of geographically-stretched households are living [4, 19, 24].

Epidemiologic research on HIV has long shown links between mobility and higher HIV prevalence; migrants have been found to have higher odds of living with HIV (ranging from 1.18–2.55) [25–28] and at the population-level, mobility has been associated with higher HIV prevalence [2, 29, 30]. Longitudinal studies have shown associations between multiple forms of mobility and HIV acquisition, which consistently have varied by sex, with women’s mobility particularly heightening their risk for HIV acquisition [2, 4, 6]. In KwaZulu-Natal, South Africa, the risk of HIV acquisition was influenced both by changing residences over longer distances or spending longer periods of time outside one’s primary residence, with differences by sex in 2000–2014: it increased by 50% for migration distances of 40 km for men and 109 km for women, and increased by 50% when participants spent 44% (men) and 90% (women) of their respective time outside their home community [31]. In the same setting, the highest risks of HIV acquisition were seen in women who migrated frequently between 2004 and 2015 [32]. In Rakai, Uganda, in which a cohort of HIV-negative adults were followed prospectively for 16 years, risk of HIV acquisition was heightened in the first two years after in-migration particularly among women (IRR = 1.92, 95% CI 1.52–2.43) compared to men (IRR = 1.52, 95% CI 0.99–2.33) [6]. Research in Rakai also found that migrants living with HIV selectively migrated to high prevalence areas, and that out-migrants from these areas geographically dispersed (rather than migrating to low prevalence areas) [33]. Geospatial modeling research focused on migration flows have helped to clarify the role of specific migration hubs and directions of human movement in the sustaining of epidemics in the region [23, 34, 35].

The complex and dynamic nature of mobility requires use of multiple measures and sex-stratified analyses to understand the influence of varied forms and patterns of mobility on HIV risks in specific populations and settings. For example, we must examine not only migration, or permanent changes in residence, but also short-term mobility, inclusive of mobility for multiple purposes (such as work and social obligations) in order to understand the full effects of mobility on HIV risk. Here, we leverage data from the Sustainable East Africa Research in Community Health (SEARCH) trial in rural Kenya and Uganda which used universal antiretroviral therapy (ART) with annual population testing and a multidisease, patient-centered strategy to reduce new HIV infections and improve community health [36]. While universal ART treatment through SEARCH did not result in significantly lower HIV incidence in the intervention vs. control arms due to national guideline changes to universal HIV treatment, population-level viral suppression was higher and HIV-associated tuberculosis (TB), mortality, and HIV vertical transmission were lower in intervention compared to control communities [36, 37]. The SEARCH intervention also increased retention in care among those living with HIV at one year [38]. Prior research from SEARCH also found associations between mobility and risk of HIV seroconversion: after adjustment for other risk factors, men who were mobile [≥ 1 month of prior year living outside community] (aRR = 1.68, 95% CI 1.09–2.60) and women who were mobile [lived > 1 month of the prior year away from the community] (aRR = 1.49; 95% CI 1.04–2.11) were more likely to seroconvert [39]. The findings suggested that in the context of universal testing and treatment programs, additional strategies were needed to address HIV acquisition risks among mobile populations. To extend this prior work, here, we examine the risk of HIV acquisition over 3 years in categories of mobile compared to non-mobile adults in the SEARCH population cohort in Kenya and Uganda, using a full range of measures of mobility at baseline and three year follow-up and examining sex differences in seroconversion risk associated with mobility.

## Methods

### Study Design and Sample

This study examined the risk of HIV acquisition over 3 years in categories of mobile compared to non-mobile adults living in 32 rural communities in three regions of Uganda and Kenya participating in the SEARCH trial. Rural communities are those that have low population density and are geographically isolated and far from major towns and cities. SEARCH (NCT01864603) was a cluster randomized trial conducted from 2013 through 2017 to test whether annual population testing and delivery of universal ART with a multidisease (HIV, diabetes, hypertension) person-centered care model could reduce new HIV infections and improve community health outcomes [40]. The detailed study protocols have been described elsewhere [36], but briefly, multidisease community health campaigns were completed in all study communities at baseline and then repeated annually in intervention communities and at the end of the trial for all communities. Intervention communities had person-centered streamlined HIV care that focused on reducing structural barriers to care and improving relationships between patients and clinics. The primary trial end point was the cumulative incidence of HIV infection (confirmed by the Bio-Rad Genius HIV 1/2 Confirmatory Assay and Western blot testing) at 3 years among residents in the baseline census. In the SEARCH incidence cohort of 117,114 adults who were at least 15 years of age, stable residents (lived in the community six or more months in the past year), and HIV-negative at baseline, there were 704 confirmed new HIV infections reported at 3 years [36].

The trial protocol received ethical approval from the Ethical Review Committee of the Kenya Medical Research Institute (KEMRI/SERU/CMR/3052); the Makerere University School of Medicine Research and Ethics Committee in Uganda (2015-040) and the Uganda National Council for Science and Technology (HS 1834); and the University of California, San Francisco Committee on Human Research (14-15058). Community-level consent (as described in the study protocol [36]) and oral informed consent from individual participants were provided for the census enumeration and the health campaigns, and written informed consent was provided in cases in which a participant was ineligible for ART on the basis of country guidelines. The trial was conducted in accordance with the principles of the Declaration of Helsinki and was conducted with oversight by a data and safety monitoring board.

### Measures of Mobility

To better understand mobility and migration in the context of the multi-year SEARCH trial, we collected data on migration in the past 12 months and nights spent away from the primary residence. Migration was defined as permanent changes of primary residence across defined geopolitical boundaries. As this was an incidence cohort, it was a closed cohort, and new in-migrants were not added to the cohort during the study. Whether study participants out-migrated or in-migrated back into the study region, we tried to gather data on the migration location. At baseline, we recorded those who had spent greater than 1 month living outside the home community in the past 12 months as well as nights spent at the main residence in the past month. At year three, we recorded those who had spent greater than 1 month living outside the home community in the past year. We also noted changes of residence in the past 12 months, and those who had out-migrated from the study community. We also collected data on mobility in the past month by asking participants how many nights they had spent at their main residence in the past month.

### Statistical Analysis

We examined HIV incidence by mobility pre-baseline and over 3 years and evaluated differences by sex. Bivariate associations were assessed using Rao-Scott-based F-tests of association adjusted for clustering on community for categorical and binary variables and cluster-adjusted Wilcoxon rank sum tests for continuous variables. Poisson regression models were used to estimate incidence rate ratios (IRRs) of HIV acquisition among categories of mobile relative to non-mobile adults, with sex-stratified multivariable models adjusted for region and demographic characteristics, and for clustering by community using a cluster-based Huber-White sandwich robust estimator. To examine heterogeneity of changes in incidence over time by region, we examined the potential for differences in effects of metrics of mobility on incidence by region in a sensitivity analysis that included inverse probability weighting to adjust for censoring due to out-migration. The Poisson models were fitted with interaction terms of mobility metric by region, with contrasts to examine the main effects of mobility of region as well as their interaction to assess the moderating effects of region. Analyses were conducted using Stata statistical software (version 16) (College Station, TX).

## Results

### Demographics

As reported previously, at baseline, the HIV incidence cohort included 64,828 women and 52,286 men (n = 117,114) (Table 1) of whom 1813 (1.5%) died, 8502 (7.3%) out-migrated, and 11,716 (10%) were not tested for HIV at year three [36]. Almost 50% of the sample was under 30 years old, with almost even percentages from Eastern Uganda (35.4%), Western Uganda (33.3%), and Kenya (31.3%). Most of the sample was single (60.5%), with more men (35.8%) than women (20.4%) reporting being married/cohabitating (p < 0.001). Most participants had completed up to secondary level education (80.3%) and reported informal low HIV risk occupations (such as farmer, shopkeeper, market vendor, hotel worker, housewife, household worker, construction worker, miner). Alcohol consumption was significantly higher among men (25.0%) compared to women (7.4%) (p < 0.001).

**Table 1:** Population demographic characteristics and mobility (HIV incidence cohort), baseline and year 3, by sex

Characteristic	Total		Women		Men		*F* test (DF)	*p* value
	N	%	N	%	N	%		
Sociodemographic characteristics at baseline								
Total adult resident population (55.4% female, 44.7% male)	117,114	100.0	64,828	100.0	52,286	100.0		
Age							24.19 (4.27, 132.38)	< 0.001
15–19	25,069	21.4	12,447	19.2	12,622	24.1		
20–24	17,711	15.1	9,928	15.3	7,783	14.9		
25–29	14,377	12.3	8,060	12.4	6,317	12.1		
30–34	11,497	9.8	6425	9.9	5072	9.7		
35–39	9457	8.1	5328	8.2	4129	7.9		
40–44	8186	7.0	4545	7.0	3641	7.0		
45–49	6205	5.3	3534	5.5	2671	5.1		
50–54	6322	5.4	3825	5.9	2497	4.8		
55–59	4235	3.6	2441	3.8	1794	3.4		
60–64	4149	3.5	2502	3.9	1,647	3.2		
65 or older	9906	8.5	5793	8.9	4113	7.9		
Region							1.64 (1.66, 51.51)	0.2076
Eastern Uganda	41,424	35.4	22,703	35.0	18,721	35.8		
Western Uganda	38,998	33.3	21,786	33.6	17,212	32.9		
Kenya	36,692	31.3	20,339	31.4	16,353	31.3		
Marital status							647.82 (2.07, 64.31)	< 0.001
Married (includes cohabiting)	33,338	28.5	13,200	20.4	20,138	38.5		
Single	70,808	60.5	40,268	62.1	30,540	58.4		
Widowed	9236	7.9	8584	13.2	652	1.3		
Separated or divorced	3732	3.2	2776	4.3	956	1.8		
Education level							158.86 (1, 31)	< 0.001
Completed up to secondary (or missing)	94,066	80.3	54,682	84.4	39,384	75.3		
Started secondary level or beyond	23,048	19.7	10,146	15.7	12,902	24.7		
Household wealth index: quantiles							49.28 (3.07, 95.15)	< 0.001
0	18,802	16.1	10,943	16.9	7859	15.1		
1	20,815	17.8	11,764	18.2	9051	17.3		
2	23,681	20.3	13,284	20.5	10,397	19.9		
3	25,709	22.0	14,020	21.7	11,689	22.4		
4	27,922	23.9	14,717	22.7	13,205	25.3		
Occupation								
Formal sector (teacher, student, gov’t worker, military worker, health worker, factory worker)	26,771	22.9	11,516	17.8	15,255	29.2	221.52 (1, 31)	< 0.001
Informal high risk (fish trader, fisherman, bar owner, bar worker, transport, tourism)	4997	4.3	1427	2.2	3570	6.8	87.51 (1, 31)	< 0.001
Informal low risk (farmer, shopkeeper, market vendor, hotel worker, housewife, household worker, construction worker, miner)	73,627	62.9	45,931	70.9	27,696	53.0	572.18 (1, 31)	< 0.001
Any alcohol consumption (“Do you drink alcohol?”)	16,701	15.4	4441	7.4	12,260	25.0	254.28 (1, 31)	< 0.001
Circumcision (men, traditional or medical)^a^					17,776	34.1		
Mobility at baseline								
Changed residence in past 12 months, baseline	1966	1.7	988	1.5	978	1.9	13.01 (1, 31)	0.0011
≥ 1 month living outside community in past 12 months, baseline	11,337	9.7	5182	8.0	6155	11.8%	66.39 (1, 31)	< 0.001
Household presence pattern^b^, past month, baseline							45.46 (3.13, 97.17)	< 0.001
0: no nights	10,517	9.0	5897	9.1	4620	8.9		
1: few nights	5100	4.4	2341	3.6	2759	5.3		
2: less than half of the month	912	0.8	388	0.6	524	1.0		
3: more than half of the month	1313	1.1	575	0.9	738	1.4		
4: most nights	19,044	16.3	9716	15.0	9328	17.9		
5: every night	79,880	68.4	45,745	70.7	34,135	65.5		
Mobility at year 3								
Out-migrated from study community (proxy report) by year 3	8502	7.3	4906	7.7	3605	7.1	4.34 (1, 31)	0.0456
Changed residence in past 12 mo., yr 3	3783	3.9	2189	4.0	1594	3.8	1.31 (1, 31)	0.2614
Spent > 6 mo. in past year outside community, year 3 (self-report)	3019	3.1	1573	2.9	1446	3.4	5.67 (1, 31)	0.0236
Spent > 12 mo. in past 3 yrs outside community, year 3 (self-report)	1046	1.1	591	1.1	455	1.1	0.02 (1, 31)	0.8932
≥ 1 month living outside community in past 12 months, year 3	5747	4.9	2655	4.1	3092	5.9	129.11 (1, 31)	< 0.001
Household presence pattern^b^, past month, year 3							60.82 (3.84, 119.11)	< 0.001
0: no nights	8020	8.2	4625	8.4	3395	8.0		
1: few nights	3184	3.3	1437	2.6	1747	4.1		
2: less than half of the month	399	0.4	147	0.3	252	0.6		
3: more than half of the month	711	0.7	277	0.5	434	1.0		
4: most nights	14,399	14.8	7212	13.2	7187	16.9		
5: every night	70,596	72.6	41,104	75.0	29,492	69.4		
Lived outside community in past 5 years, year 3	1395	1.4	884	1.6	511	1.2	20.89 (1, 31)	0.0001

Data in Table 1 are shown for the study HIV Incidence Cohort, the cohort of 117,114 adults living in SEARCH communities who were at least 15 years of age, stable residents, and HIV-negative at baseline (2013–2014). Data are column percentages. *p* = Sex differences in proportions using Rao–Scott-based F tests of association, adjusted for clustering on community using a cluster-based Huber-White sandwich robust estimator

^a^15.5% of men report ritual circumcision, 18.6% report medical circumcision

^b^Question: How many nights did you spend at your main residence in the past month?

### Mobility at Baseline and Year 3

**Fig. 1:**
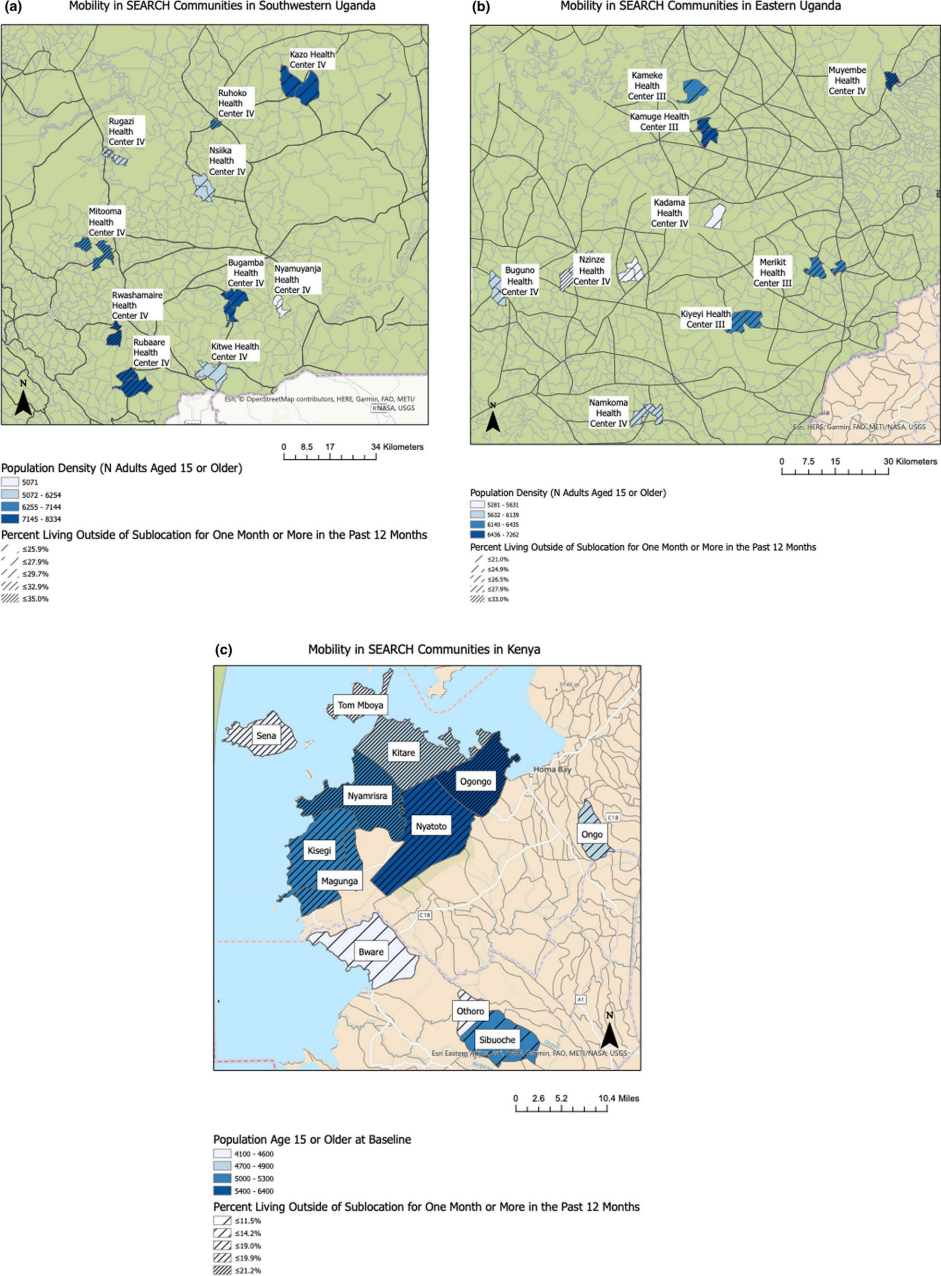
(a–c Maps of study communities by magnitude of population and past-year mobility at baseline, in Southwestern Uganda, Eastern Uganda, and Western Kenya. Notes: Fig. 1a-c shows adult population density (n of adults) and proportion of total adult population who lived outside community 1 or more months in past 12 months (at study baseline, 2013–2014), for each study community, by region

Figure 1A–C display the magnitude of population size and past year mobility across the three regions. At baseline, 11,337 adults (9.7%) reported having lived at least 1 month outside of their community in the past 12 months (8.0% of women and 11.8% of men, *p* < 0.001) (Table 1). At year three, 5747 adults remaining in cohort (4.9%) reported having lived at least 1 month outside of their community in past 12 months (4.1% of women and 5.9% of men, *p* < 0.001). At baseline, 10,517 adults (9.0%) reported not having spent any nights at their primary residence in the past month while 79,880 adults (68.4%) had spent every night in their residence in past month. At baseline, 36,886 (31.6%) spent at least some nights away in the past month (29.3% of women and 34.5% of men, *p* < 0.001) (data not shown). At year three, 26,713 adults (27.5%) spent at least some nights away from their residence in the past month (25.0% of women and 30.6% of men, *p* < 0.001) (data not shown). By year three, 8502 members of the incidence cohort (7.3%) had out-migrated (7.7% of women and 7.1% of men, *p* = 0.046).

### Risk of HIV Acquisition At Baseline and Year 3 by Mobility Status and Sex

**Fig. 2:**
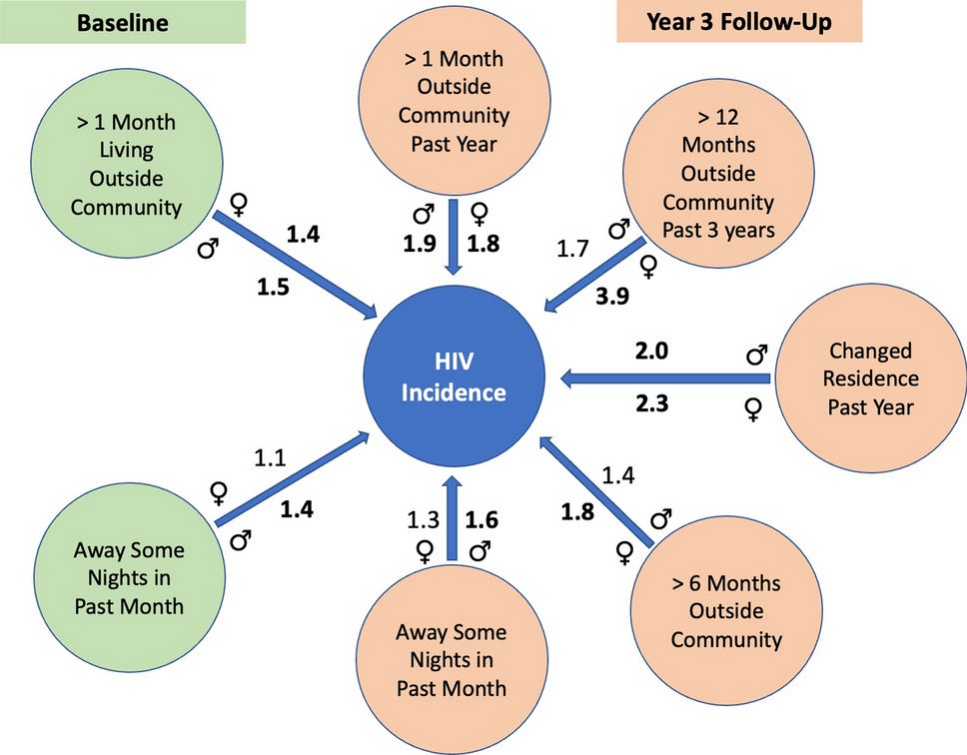
Risk of HIV acquisition, by measures of mobility and sex. Note: Numbers beside arrows refer to the sex-specific adjusted relative risk of HIV seroconversion over the 3-year period adjusted for age, region, marital status, education level, household wealth index, occupation category, alcohol use, and circumcision (in men), and adjusted for clustering by community. Bolded IRRs are statistically significant ay the p < 0.05 level

The risk of HIV acquisition was 47% higher among all people who reported living ≥ 1 month outside their community in the past 12 months prior to the baseline visit (Adj IRR = 1.47, 95% CI 1.18–1.82), with similar effects for women and men (Fig. 2, Table 2). In addition, the risk of seroconversion was 1.9 times higher in those who lived ≥ 1 month outside their community in the past 12 months prior to the year three visit (Adj IRR = 1.88, 95% CI 1.48–2.38), with similar effects for women and men (Table 2). While the risk of HIV acquisition was not significant among all people who spent some nights away in the past month at baseline (Adj IRR = 1.17, 95% CI 0.99–1.40), it was 42% higher among men who spent some nights away in the past month (Adj IRR = 1.42, 95% CI 1.11–1.81). The association for women was not significant for this baseline measure (Adj IRR = 1.06, 95% CI 0.85–1.32). At the year three visit, the risk of HIV acquisition was 41% higher among all adults who spent some nights away in the past month (Adj IRR = 1.41, 95% CI 1.12–1.78), with this association being significant for men (Adj IRR = 1.59, 95% CI 1.17–2.17) but not women (Adj IRR = 1.27, 95% CI 0.92–1.75). Furthermore, the risk of HIV acquisition was higher among the small proportion of the population who had spent more than 12 months of the past 3 years outside of the community (n = 1046, 1.1%), as reported at the year three visit (Adj IRR = 3.20, 95% CI 2.21–4.64). This greater than 12 months effect in the pooled model was driven by differences among women (Adj IRR = 3.90, 95% CI 2.63–5.78) and was not significant in men (n = 455, Adj IRR = 1.68, 95% CI 0.75–3.80). Similarly, the risk of seroconversion was higher for women but not men who reported spending more than 6 months of the past year outside of the community at the year three visit (Adj IRR = 1.84, 95% CI 1.23–2.76), although this was not significant in men (Adj IRR = 1.35, 95% CI 0.83–2.19).

**Table 2:** Risk of HIV acquisition associated with forms of mobility, incidence cohort (n = 117,114), and by sex

Measures of mobility at baseline and year 3	Adjusted relative risk of HIV seroconversion^a^ over 3-year period											
	Total population				Women				Men			
	Adj IRR	*p* value	95% CI		Adj IRR	*p* value	95% CI		Adj IRR	*p* value	95% CI	
** ≥ **1 mo. living outside community, past 12 mo., baseline	1.47	0.001	1.18	1.82	1.41	0.038	1.02	1.95	1.49	0.013	1.09	2.05
Away at least some nights in past mo., baseline	1.17	0.070	0.99	1.40	1.06	0.627	0.85	1.32	1.42	0.006	1.11	1.81
Spent > 6 mo. in past yr outside community, yr 3	1.70	0.001	1.24	2.34	1.84	0.003	1.23	2.76	1.35	0.235	0.83	2.19
Spent > 12 mo. in past 3 yrs outside community, yr 3	3.20	< 0.001	2.21	4.64	3.90	< 0.001	2.63	5.78	1.68	0.209	0.75	3.80
** ≥ **1 mo. living outside community, past 12 mo., yr 3	1.88	< 0.001	1.48	2.38	1.83	0.001	1.27	2.64	1.85	0.001	1.31	2.60
Changed residence in past 12 mo., yr 3	2.30	< 0.001	1.80	2.95	2.28	< 0.001	1.68	3.09	2.03	0.016	1.14	3.61
Away at least some nights in past mo., yr 3	1.41	0.004	1.12	1.78	1.27	0.152	0.92	1.75	1.59	0.003	1.17	2.17
Lived outside community in past 5 yrs, yr 3	1.78	0.005	1.19	2.66	1.82	0.014	1.13	2.92	1.65	0.284	0.66	4.16

Poisson regression models adjusted for sex (in pooled models), age, region, marital status, education level, household wealth index, occupation category, alcohol use, and circumcision (men only). Adjusted for clustering by community using a cluster-based Huber-White sandwich robust estimator

^a^Confirmed to be HIV-positive  through rapid testing, Geenius, and Western blot at follow up year 3

There were no significant interaction effects of mobility by SEARCH study arm; the effects of mobility on seroconversion risk at the individual level were similar across both study arms (Suppl. Table 1). In sensitivity analyses examining the risk of HIV acquisition associated with forms of mobility by sex (adjusted for clustering in communities, and with inverse probability weighting to adjust for censoring due to out-migration), men and women exhibited similar risks of HIV seroconversion when spending about 1 month outside their home community in the past year at both baseline and year three visits (Suppl. Table 2). However, women showed increased risks of HIV acquisition with greater periods of time spent outside their home community, while the effect was not significant for men. For example, women who spent more than 6 months in the past year outside community prior to the year three visit (Adj IRR = 1.84, 95% CI 1.23–2.75), those who spent more than 12 months in the past 3 years outside of community at year three (Adj IRR = 3.76, 95% CI 2.45–5.78), and those who changed residence in the past 12 months at year three (Adj IRR = 2.25, 95% CI 1.70–2.97) all had higher risks of HIV acquisition. In addition, there was no significant interaction of mobility with region (Χ^2^(2) = 0.49, p = 0.78). However, there was a significant ‘nights away from home’ by region interaction (Χ^2^(2) = 9.51, p < 0.01), such that a higher HIV incidence was observed in mobile adults in Kenya and Western Uganda relative to those in Eastern Uganda (Suppl. Table 3; Suppl. Figure 1).

## Discussion

In this population-based study in rural East African communities, mobility was common among rural residents defined as “stable” at study baseline. While the incidence cohort was limited to adults who had lived at least 6 months of the past year in their primary residence, a small proportion were away for a significant period of time, with 10% having reported 1 month away from their home community at baseline. By year three, 7% had out-migrated. More mobile residents faced increased risk for HIV acquisition based on the time spent away, with those who lived more than 1 month outside their home community showing higher odds of seroconversion at baseline and at year three. HIV risk was highest among those who spent more than 12 months of the past 3 years outside of the community and for those who spent more than 6 months of the past year outside of the community at year three. Prior research has shown heightened HIV risks among female relative to male migrants [3, 19, 41]. Here, we observe the effects of some forms of mobility on HIV acquisition risk among women, but not among men.

Notably, even in rural East African settings, mobility is commonplace and driven by underlying labor market conditions. In contexts of high unemployment in the formal sector, typically, individuals and households pursue diverse opportunities for livelihood strategies that may be sporadic and varied across locations and over time [5, 42, 43]. Mobility is also driven by social ties of obligation (as when individuals travel to family members’ homes to care for them when sick) and cultural obligations for the maintenance of land, familial lineage, and community (as when individuals travel for weddings, funerals, after births, harvesting, and other important community events, including participating in elections) [44–46]. Studies have shown that compared with non-mobile individuals, mobile people are at an increased risk of acquiring HIV in part because of a propensity towards higher-risk sexual behavior and in part because of increased exposure risk due to their mobility, i.e. characteristics of destinations (such as HIV prevalence) [3, 4, 32, 41, 47–49]. Those who are mobile may be relocating or traveling because of higher-risk situations at their place of origin, and mobility can increase potential risk exposure at destinations. Mobile individuals have often experienced life disruptions such as the death of a spouse, or marital separation, all of which heighten their vulnerability and separates them from their social networks [4, 42]. In the host community, they get exposed to a new environment and are covered by anonymity, therefore increasing the likelihood of riskier sexual behavior [2, 50]. A proportion of the ‘at risk’ population out-migrated and potentially may have been behaviorally at higher risk of HIV acquisition relative to more stable residents, at least by virtue of age: mean age of out-migrants was 25.5 compared to a mean age of 35.4 among those who remained in communities over the period. Nevertheless, mobility among the marginally more stable population was also associated with the risk of HIV seroconversion by year three, across several measures taken both at baseline and year three.

While the temporal ordering of exposure and outcome cannot be definitively ascertained for mobility reported at the year three visit, the magnitude of association for HIV incidence was highest for those who had lived more than 12 months in the past 3 years outside the community and those who had changed residence in the past 12 months. This finding is in line with studies from South Africa and Uganda documenting increased HIV acquisition risk among mobile adults [6, 31, 51]. Findings from a population-based cohort study conducted in KwaZulu-Natal, South Africa show that even in the context of ART provision, the incidence rate among a group of highly mobile adults was three times higher compared to those who migrated with less intensity [51].

At the population level, studies in sub-Saharan Africa since 2020 show that mobility leads to complex networks of HIV risk flows that show substantial geographical variation [52, 53]. Mobility-driven transmission frequently occurs such that communities import and export HIV [23, 52, 54]. A study in Namibia found that 40% of risk was mobility-driven and that networks contained multiple risk hubs [53]. In Uganda it was found that these high HIV risk hubs preferentially attracted young, high-prevalence migrants with untreated HIV infection who come from diverse populations [33] but out-migration from these “hot spots” infrequently contributes to cross-community HIV transmission in destinations [33, 54]. In particular, young, mobile men are difficult to maintain in care and have been found to be persistently viremic in Ugandan universal testing and treatment studies [12], showing just how hard it is to make progress with mobile populations.

While mobility-driven risk flows underlie generalized HIV epidemics in sub-Saharan Africa and challenge HIV control efforts [53], to-date, few but promising global elimination strategies have considered mobility in prevention and treatment approaches [55–57]. A randomized trial in Kenya and Uganda among mobile people living with HIV tested a patient-centered intervention that included dynamic choice of a "travel pack" (emergency ART supply, discrete ART packaging, and travel checklist), facilitated transfer to out-of-community clinics, multi-month and offsite refills, and hotline access to a mobility coordinator. The intervention improved retention in care (risk ratio: 1.06 [1.02–1.1]; p < 0.001) and ART possession (risk ratio: 1.07 [1.03–1.11]; p < 0.001), with larger effect sizes among persons with baseline non-suppression and high mobility. Another person-centered HIV prevention delivery model was tested in Kenya and Uganda among a highly mobile population and tested a dynamic choice HIV prevention (DCP) intervention for persons at risk of HIV. That model was responsive to varying personal preferences over time and key components included flexibility and responsiveness to client desires and choices for pre-exposure prophylaxis [PrEP]/post-exposure prophylaxis [PEP], clinic vs. off-site visits, self- or clinician-based HIV testing, and client and staff feedback. In South Africa, a smartphone app that used GPS location data was developed to characterize the mobility of pregnant and postpartum women living with HIV to improve HIV care engagement. Unfortunately, challenges with inconsistent smart phone access and data impeded the feasibility of that study. Taken together, these studies suggest that patient-centered interventions that include dynamic choice components may be especially promising and relevant for mobile populations.

Some associations between mobility and HIV incidence were significant for women but not men, or for men but not women, adding to a growing literature that highlights the gendered differences in mobility [2, 4, 19, 58]. These sex differences in HIV incidence rates and mobility, with effects for women but not men who spent time living outside the community, as reported in year three, are novel. Analyses using SEARCH study data in 2021 found that overall, women had a higher risk of sero-conversion compared to men. However, while both men and women who were mobile (spent > 1 month outside the home community in the past year) had an increased incidence of sero-conversion, the point estimate was higher for men (aRR 1.68; 95% CI 1.09–2.60) compared to women (aRR 1.49; 95% CI 1.04–2.11) [39]. However, in a couples study in Kenya, the mobility of women, but not men, was associated with HIV infection: women who were mobile and had non-mobile spouses had two times the likelihood of HIV infection compared to individuals in couples where both the man and woman were non-mobile [3].

It is possible that extended time spent outside the community is significantly associated with increased HIV incidence for women, but not men, because of the gendered social norms that influence the lives of men and women in this setting [3]. While we do not have detailed information as part of this incidence cohort about reasons for travel, among a subset from the larger trial population, the predominant reasons for why men travelled in Kenya were related to taking part in construction work (33.6%), mobility related to the fish trade (27.9%), and for attending funerals (42.4%) [41] In that same study, women in Kenya travelled predominantly for market trading (83.5%), mobility related to the fish trade (11.1%), and attending funerals (50.1%) [41]. In this study, we saw elevated odds of HIV infection in men who spent at least some nights away in the past month at both baseline and 3 years. It is possible that these men engaged in higher-risk sexual practices when they were travelling. Studies suggest that male migrants have more concurrent and casual partners [32, 41, 58] and this may lead to the increase in HIV incidence among men who report shorter trips. While men may engage in higher-risk behaviors with casual or concurrent partners, migrant women engage in more transactional sex to meet subsistence needs [4, 43], and these relationships, with very unequal power dynamics, may mean that women engage in unsafe sexual practices because they are unable to negotiate for prevention methods, such as condom or PrEP use. Men’s engagement in higher-risk sexual practices such as having multiple sexual partners is seen as normative, thus while mobility may increase opportunities to have more partners, men may not socially require the anonymity of travel to engage in multiple sexual relationships. In contrast, women are expected to be monogamous and their engagement in extra-marital partnerships is stigmatized, causing women to hide extra-marital partnerships from their husbands and communities [3]. Thus, mobility for women both offers opportunities for more partnerships and also the social protection of anonymity and freedom from the social monitoring that occurs in home communities [4]. The act of hiding these activities also may mean that women do not seek out services to protect themselves from HIV infection. More research that further explores associations between forms of mobility and HIV acquisition risks that differ by sex are needed.

This study has some limitations. While the incidence cohort was composed to be residentially stable, 7.3% of the cohort out-migrated by year three, showing that a significant portion of the population was spending considerable time away from their home community. In addition, those who out-migrated were likely to have been at higher risk of HIV compared to stable residents. Therefore, our estimates may under-report the true risk of sero-conversion by year three. Universal test and treat (UTT) trials have demonstrated a higher risk for HIV incident infection and lack of viral suppression among mobile residents compared to non-mobile residents [36, 59–61] and labor-related mobility has been associated with living with HIV and higher-risk sexual behaviors, such as relationship concurrency, among both men and women [2, 41]. The unavoidable characteristics of universal test and treat trials can dilute the differences seen between study arms such that the effect of the interventions on HIV incidence is underestimated [62]. In particular, intervention effects are likely diluted by migration both into and out of the study communities [63]. Also, at year three, 10% of the sample were not tested for HIV. It is possible that those who were lost to follow-up or did not test may also be higher-risk for HIV acquisition, and so again, our findings may under-estimate the true risk of sero-conversion. In addition, the temporal dimensions of population mobility are important. While we are able to present data over time, the data are cross-sectional point prevalences of mobility at two timepoints. We are unable to establish the temporal ordering of exposure and outcome at year three. Finally, limited sample size may have led to insufficient power to conduct some sex-specific analyses. Therefore, a lack of statistical significance in some analyses may be due to sample size and should not be considered as evidence of heterogeneity by sex. These limitations notwithstanding, these data support a growing body of longitudinal studies that emphasize that population mobility and HIV epidemics are linked via multiple pathways and that a continued focus on the HIV prevention and care needs among mobile individuals is warranted.

## Conclusions

Mobility is significantly associated with the risk of HIV acquisition in rural Kenya and Uganda. Furthermore, the association between mobility on HIV incidence is influenced by both sex and the duration and form of mobility. Mobility can lead to a marked redistribution of population and HIV risk, changing the location of high HIV transmission areas and creating new transmission corridors that can have a substantial effect on HIV epidemics locally and on HIV elimination efforts more broadly. Novel approaches that adapt prevention strategies and care programs specifically for mobile populations are crucial for achieving the UNAIDS Sustainable Development Goal to end the epidemic by 2030.

## Supplementary Information

Below is the link to the electronic supplementary material.

Supplementary file1 (DOCX 283 KB)

## Data Availability

Data are available from the authors upon reasonable request.
