# Supplementary material for: Metrics of Mobility by Sex are Associated with HIV Incidence in Rural Kenya and Uganda
Source: AIDS Behav. 2025 May 6;29(9):2869–81. doi: 10.1007/s10461-025-04743-6 (PMC12229744; doi:10.1007/s10461-025-04743-6)
Supplement: Supplementary file 1 — Supplementary file1 (DOCX 283 KB) [file 10461_2025_4743_MOESM1_ESM.docx]

**Supplementary Table 1: Adjusted relative risk of HIV seroconversion, incidence cohort (n=704), by measures of mobility and study arm**

| **Measures of mobility at baseline and year 3** | **Adjusted relative risk of HIV seroconversion over 3-year period** | | | | | | | |
| --- | --- | --- | --- | --- | --- | --- | --- | --- |
|  | **Control Arm** | | | | **Intervention Arm** | | | |
|  | **Adj IRR** | ***p-value*** | **95% CI** |  | **Adj IRR** | ***p-value*** | **95% CI** | |
| >=1 mo. living outside community, past 12 mo., baseline | 1.47 | 0.014 | 1.08 | 2.00 | 1.45 | 0.021 | 1.06 | 1.99 |
| Away at least some nights in past mo., baseline | 1.04 | 0.808 | 0.777 | 1.38 | 1.31 | 0.004 | 1.09 | 1.58 |
| Spent >6 mo. in past year outside community, year 3 | 1.65 | 0.057 | 0.985 | 2.77 | 1.74 | 0.009 | 1.15 | 2.64 |
| Spent >12 mo. in past 3 years outside community, year 3 | 4.02 | <0.001 | 2.594 | 6.22 | 2.29 | 0.011 | 1.21 | 4.31 |
| >=1 mo. living outside community, past 12 mo., year 3 | 1.72 | 0.009 | 1.148 | 2.59 | 2.00 | <0.001 | 1.50 | 2.67 |
| Changed residence in past 12 mo., year 3 | 2.57 | <0.001 | 1.870 | 3.52 | 2.05 | <0.001 | 1.45 | 2.90 |
| Away at least some nights in past mo., year 3 | 1.37 | 0.088 | 0.953 | 1.98 | 1.44 | 0.018 | 1.06 | 1.96 |
| Lived outside community in past 5 years, year 3 | 1.58 | 0.179 | 0.810 | 3.09 | 2.03 | 0.003 | 1.28 | 3.24 |

Note: Poisson regression models adjusted for sex (in pooled models), age, region, marital status, education level, household wealth index, occupation category, alcohol use, and circumcision (men only). Adjusted for clustering by community using a cluster-based Huber-White sandwich robust estimator.

* Confirmed to be HIV+ through rapid testing, Geenius, and Western blot at follow up year 3.

**Supplementary Table 2: Sensitivity analysis: Risk of HIV acquisition associated with forms of mobility, incidence cohort (n=704), and by sex (adjusted for clustering in communities, and with inverse probability weighting to adjust for censoring due to out-migration)**

| **Measures of mobility at baseline and year 3** | **Adjusted relative risk of HIV seroconversion over 3-year period** | | | | | | | | | | | | | |  |
| --- | --- | --- | --- | --- | --- | --- | --- | --- | --- | --- | --- | --- | --- | --- | --- |
|  | **Total population** | | | | **Women** | | | | | **Men** | | | | |  |
|  | **Adj IRR** | ***p-value*** | **95% CI** | | | **Adj IRR** | ***p-value*** | **95% CI** | | | **Adj IRR** | ***p-value*** | **95% CI** | | |
| ≥1 mo. living outside community, past 12 mo., baseline | 1.74 | <0.001 | 1.33 | 2.27 | | 1.78 | 0.006 | 1.18 | 2.68 | | 1.73 | 0.003 | 1.20 | 2.49 | |
| Away at least some nights in past month, baseline | 1.12 | 0.336 | 0.89 | 1.41 | | 1.01 | 0.922 | 0.77 | 1.33 | | 1.31 | 0.130 | 0.92 | 1.86 | |
| Spent >6 mo. in past year outside community, year 3 | 1.73 | 0.001 | 1.27 | 2.37 | | 1.84 | 0.003 | 1.23 | 2.75 | | 1.47 | 0.199 | 0.82 | 2.63 | |
| Spent >12 mo. in past 3 yrs outside community, year 3 | 2.88 | <0.001 | 1.89 | 4.39 | | 3.76 | <0.001 | 2.45 | 5.78 | | 1.43 | 0.427 | 0.59 | 3.44 | |
| ≥1 mo. living outside community, past 12 mo., year 3 | 2.07 | <0.001 | 1.60 | 2.67 | | 2.10 | <0.001 | 1.42 | 3.11 | | 2.02 | 0.001 | 1.35 | 3.02 | |
| Changed residence in past 12 months, year 3 | 2.21 | <0.001 | 1.74 | 2.80 | | 2.25 | <0.001 | 1.70 | 2.97 | | 1.73 | 0.080 | 0.94 | 3.21 | |
| Away at least some nights in past month, year 3 | 1.35 | 0.037 | 1.02 | 1.78 | | 1.28 | 0.160 | 0.91 | 1.81 | | 1.38 | 0.121 | 0.92 | 2.08 | |
| Lived outside community in past 5 years, year 3 | 2.15 | 0.005 | 1.25 | 3.67 | | 1.93 | 0.013 | 1.15 | 3.25 | | 2.58 | 0.112 | 0.80 | 8.32 | |

Notes: Poisson regression models adjusted for sex (in pooled models), age, region, marital status, education level, household wealth index, occupation category, and circumcision (men only). Adjusted for clustering by community using a cluster-based Huber-White sandwich robust estimator. Adjusted for selection due to censoring/outmigration using inverse probability weighting.

**Supplementary Table 3: Adjusted relative risk of HIV seroconversion*, incidence cohort (n=704), by measures of mobility (full model output)**

| **Characteristic** | **Model 1: >=1 month living outside community in past 12 months, baseline** | | | | **Model 2: Away at least some nights in past month, baseline** | | | | **Model 3: Spent >6 months in past 12 months outside community, year 3** | | | | **Model 4: Spent >12 months in past 3 years outside community, year 3** | | | |
| --- | --- | --- | --- | --- | --- | --- | --- | --- | --- | --- | --- | --- | --- | --- | --- | --- |
|  | **IRR** | ***p-value*** | **95% CI** | | **IRR** | ***p-value*** | **95% CI** | | **IRR** | ***p-value*** | **95% CI** | | **IRR** | ***p-value*** | **95% CI** | |
| *Mobility* |  |  |  |  |  |  |  |  |  |  |  |  |  |  |  |  |
| >=1 months living outside community, past 12 months, baseline | 1.47 | 0.001 | 1.18 | 1.82 |  |  |  |  |  |  |  |  |  |  |  |  |
| Away at least some nights in past month, baseline |  |  |  |  | 1.17 | 0.070 | 0.99 | 1.40 |  |  |  |  |  |  |  |  |
| Spent >6 mo. in past year outside community, year 3 |  |  |  |  |  |  |  |  | 1.70 | 0.001 | 1.24 | 2.34 |  |  |  |  |
| Spent >12 mo. in past 3 years outside community, year 3 |  |  |  |  |  |  |  |  |  |  |  |  | 3.20 | <0.001 | 2.21 | 4.64 |
| *Baseline sociodemographic* |  |  |  |  |  |  |  |  |  |  |  |  |  |  |  |  |
| Sex: male (ref.: female) | 0.62 | <0.001 | 0.51 | 0.75 | 0.62 | 0<0.001 | 0.49 | 0.79 | 0.64 | <0.001 | 0.50 | 0.81 | 0.64 | <0.001 | 0.50 | 0.81 |
| Age | 0.96 | <0.001 | 0.96 | 0.97 | 0.96 | <0.001 | 0.96 | 0.97 | 0.97 | <0.001 | 0.96 | 0.97 | 0.97 | <0.001 | 0.96 | 0.97 |
| Region (ref.: E Uganda) |  |  |  |  |  |  |  |  |  |  |  |  |  |  |  |  |
| Kenya | 3.80 | <0.001 | 3.02 | 4.78 | 3.65 | <0.001 | 2.58 | 5.17 | 3.70 | <0.001 | 2.62 | 5.24 | 3.78 | <0.001 | 2.68 | 5.33 |
| Western Uganda | 2.27 | <0.001 | 1.82 | 2.83 | 2.25 | <0.001 | 1.59 | 3.18 | 2.24 | <0.001 | 1.59 | 3.16 | 2.26 | <0.001 | 1.61 | 3.18 |
| Single (ref: married, divorced, separated, widowed) | 1.34 | 0.004 | 1.04 | 1.74 | 1.35 | 0.003 | 1.11 | 1.65 | 1.31 | 0.010 | 1.07 | 1.60 | 1.29 | 0.015 | 1.05 | 1.58 |
| Secondary educ. or beyond (ref.: up to secondary) | 1.00 | 0.977 | 0.81 | 1.24 | 1.03 | 0.832 | 0.80 | 1.33 | 1.01 | 0.925 | 0.79 | 1.30 | 1.02 | 0.891 | 0.79 | 1.31 |
| Household wealth index: quartiles | 0.91 | 0.001 | 0.86 | 0.96 | 0.91 | 0.002 | 0.86 | 0.97 | 0.91 | 0.002 | 0.86 | 0.97 | 0.92 | 0.003 | 0.86 | 0.97 |
| Formal higher risk occupation | 0.25 | <0.001 | 0.18 | 0.34 | 0.25 | <0.001 | 0.18 | 0.33 | 0.25 | <0.001 | 0.19 | 0.34 | 0.26 | <0.001 | 0.19 | 0.35 |
| Informal higher risk occupation | 1.15 | 0.500 | 0.82 | 1.63 | 1.14 | 0.522 | 0.76 | 1.72 | 1.13 | 0.545 | 0.75 | 1.71 | 1.14 | 0.524 | 0.76 | 1.72 |
| Informal lower risk occupation | 0.75 | 0.034 | 0.59 | 0.96 | 0.75 | 0.028 | 0.57 | 0.97 | 0.76 | 0.034 | 0.59 | 0.98 | 0.77 | 0.034 | 0.60 | 0.98 |
| Any alcohol consumption (ref.: none) | 1.81 | <0.001 | 1.44 | 2.26 | 1.80 | <0.001 | 1.43 | 2.28 | 1.82 | <0.001 | 1.44 | 2.29 | 1.81 | <0.001 | 1.43 | 2.29 |

*Supplementary Table 3 continued on next page*

**Supplementary Table 3, continued:**

| **Characteristic** | **Model 5: >=1 month living outside community in past 12 months, year 3** | | | | **Model 6: Changed residence in past 12 months, year 3** | | | | **Model 7: Away at least some nights in past month, year 3** | | | | **Model 8: Lived outside community in past 5 years, year 3** | | | |
| --- | --- | --- | --- | --- | --- | --- | --- | --- | --- | --- | --- | --- | --- | --- | --- | --- |
|  | **IRR** | ***p-value*** | **95% CI** | | **IRR** | ***p-value*** | **95% CI** | | **IRR** | ***p-value*** | **95% CI** | | **IRR** | ***p-value*** | **95% CI** | |
| *Mobility* |  |  |  |  |  |  |  |  |  |  |  |  |  |  |  |  |
| >=1 month living outside community, past 12 months, year 3 | 1.88 | <0.001 | 1.48 | 2.38 |  |  |  |  |  |  |  |  |  |  |  |  |
| Changed residence in past 12 months, year 3 |  |  |  |  | 2.30 | <0.001 | 1.80 | 2.95 |  |  |  |  |  |  |  |  |
| Away at least some nights in past mo., yr 3 |  |  |  |  |  |  |  |  | 1.41 | 0.004 | 1.12 | 1.78 |  |  |  |  |
| Lived outside community in past 5 yrs, yr 3 |  |  |  |  |  |  |  |  |  |  |  |  | 1.78 | 0.005 | 1.19 | 2.66 |
| *Baseline sociodemographic* |  |  |  |  |  |  |  |  |  |  |  |  |  |  |  |  |
| Sex: male (ref.: female) | 0.63 | <0.001 | 0.50 | 0.80 | 0.65 | <0.001 | 0.51 | 0.82 | 0.63 | <0.001 | 0.49 | 0.79 | 0.63 | <0.001 | 0.50 | 0.81 |
| Age | 0.97 | <0.001 | 0.96 | 0.97 | 0.97 | <0.001 | 0.96 | 0.97 | 0.97 | <0.001 | 0.96 | 0.97 | 0.96 | <0.001 | 0.96 | 0.97 |
| Region (ref.: E Uganda) |  |  |  |  |  |  |  |  |  |  |  |  |  |  |  |  |
| Kenya | 3.63 | <0.001 | 2.55 | 5.16 | 3.78 | <0.001 | 2.68 | 5.34 | 3.50 | <0.001 | 2.47 | 4.95 | 3.72 | <0.001 | 2.64 | 5.23 |
| Western Uganda | 2.19 | <0.001 | 1.54 | 3.10 | 2.22 | <0.001 | 1.58 | 3.13 | 2.32 | <0.001 | 1.64 | 3.27 | 2.27 | <0.001 | 1.61 | 3.19 |
| Single (ref: married, divorced, separated, widowed) | 1.28 | 0.022 | 1.04 | 1.57 | 1.27 | 0.019 | 1.04 | 1.56 | 1.31 | 0.007 | 1.08 | 1.60 | 1.35 | 0.002 | 1.11 | 1.63 |
| Secondary educ. or beyond (ref. up to secondary) | 0.99 | 0.961 | 0.77 | 1.29 | 1.01 | 0.952 | 0.78 | 1.30 | 1.01 | 0.919 | 0.79 | 1.31 | 1.03 | 0.803 | 0.80 | 1.33 |
| Household wealth index: quartiles | 0.91 | 0.002 | 0.86 | 0.97 | 0.92 | 0.003 | 0.86 | 0.97 | 0.91 | 0.003 | 0.86 | 0.97 | 0.91 | 0.002 | 0.86 | 0.97 |
| Formal higher risk occupation | 0.25 | <0.001 | 0.18 | 0.33 | 0.25 | <0.001 | 0.19 | 0.34 | 0.24 | <0.001 | 0.18 | 0.33 | 0.25 | <0.001 | 0.19 | 0.34 |
| Informal higher risk occupation | 1.18 | 0.423 | 0.78 | 1.78 | 1.14 | 0.548 | 0.75 | 1.72 | 1.11 | 0.622 | 0.73 | 1.69 | 1.13 | 0.573 | 0.75 | 1.70 |
| Informal lower risk occupation | 0.76 | 0.045 | 0.59 | 0.99 | 0.77 | 0.041 | 0.60 | 0.99 | 0.76 | 0.029 | 0.59 | 0.97 | 0.75 | 0.027 | 0.59 | 0.97 |
| Any alcohol consumption (ref.: none) | 1.81 | <0.001 | 1.43 | 2.29 | 1.81 | <0.001 | 1.44 | 2.29 | 1.81 | <0.001 | 1.43 | 2.29 | 1.82 | <0.001 | 1.44 | 2.29 |

Note: Poisson regression models, adjusted for clustering by community using a cluster-based Huber-White sandwich robust estimator.

* Confirmed to be HIV+ through rapid testing, Geenius, and Western blot at follow up year 3.

**Supplementary Figure 1 Margin plots: Region significantly moderates effects of selected mobility metrics on HIV incidence**

**
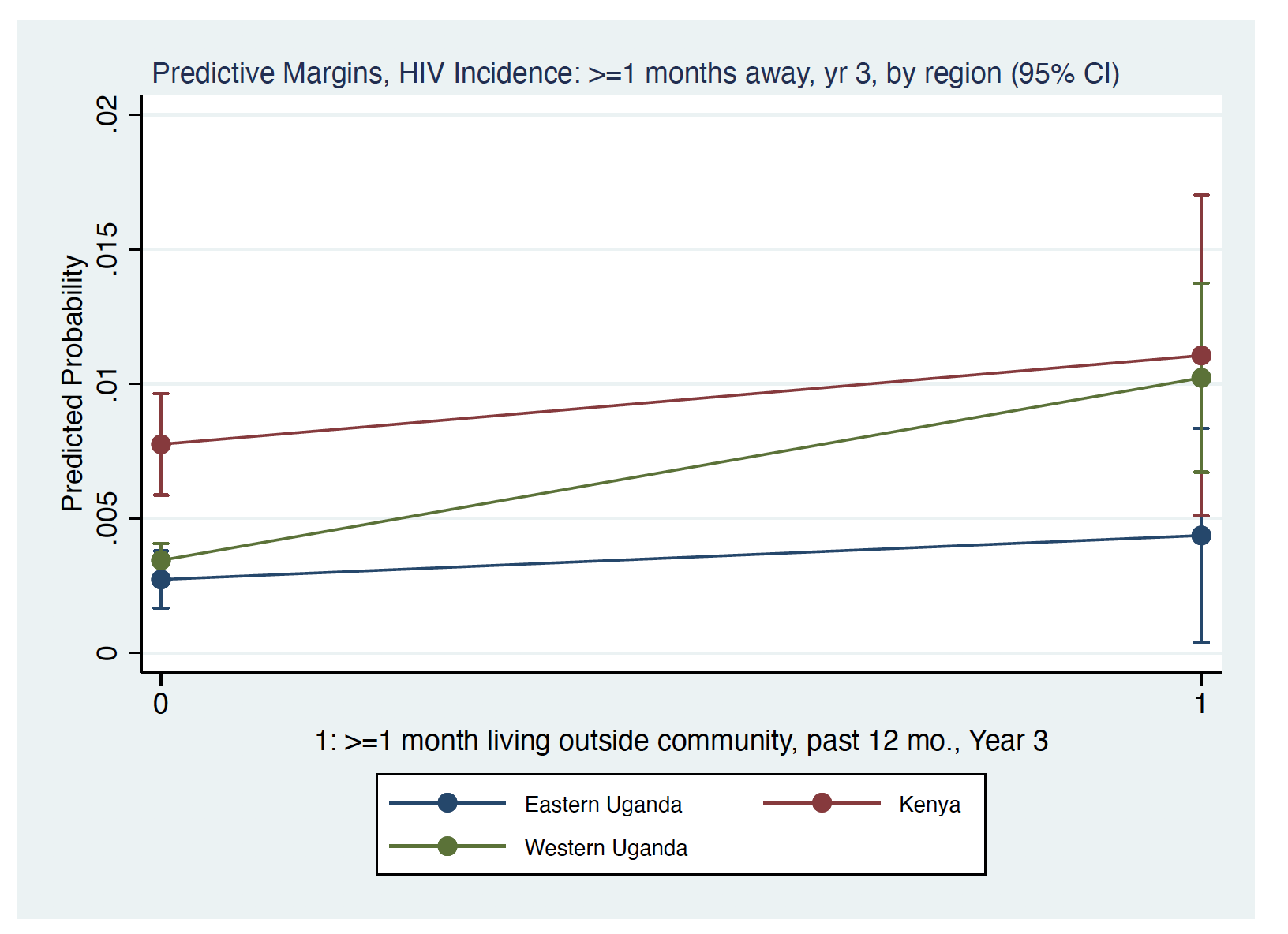

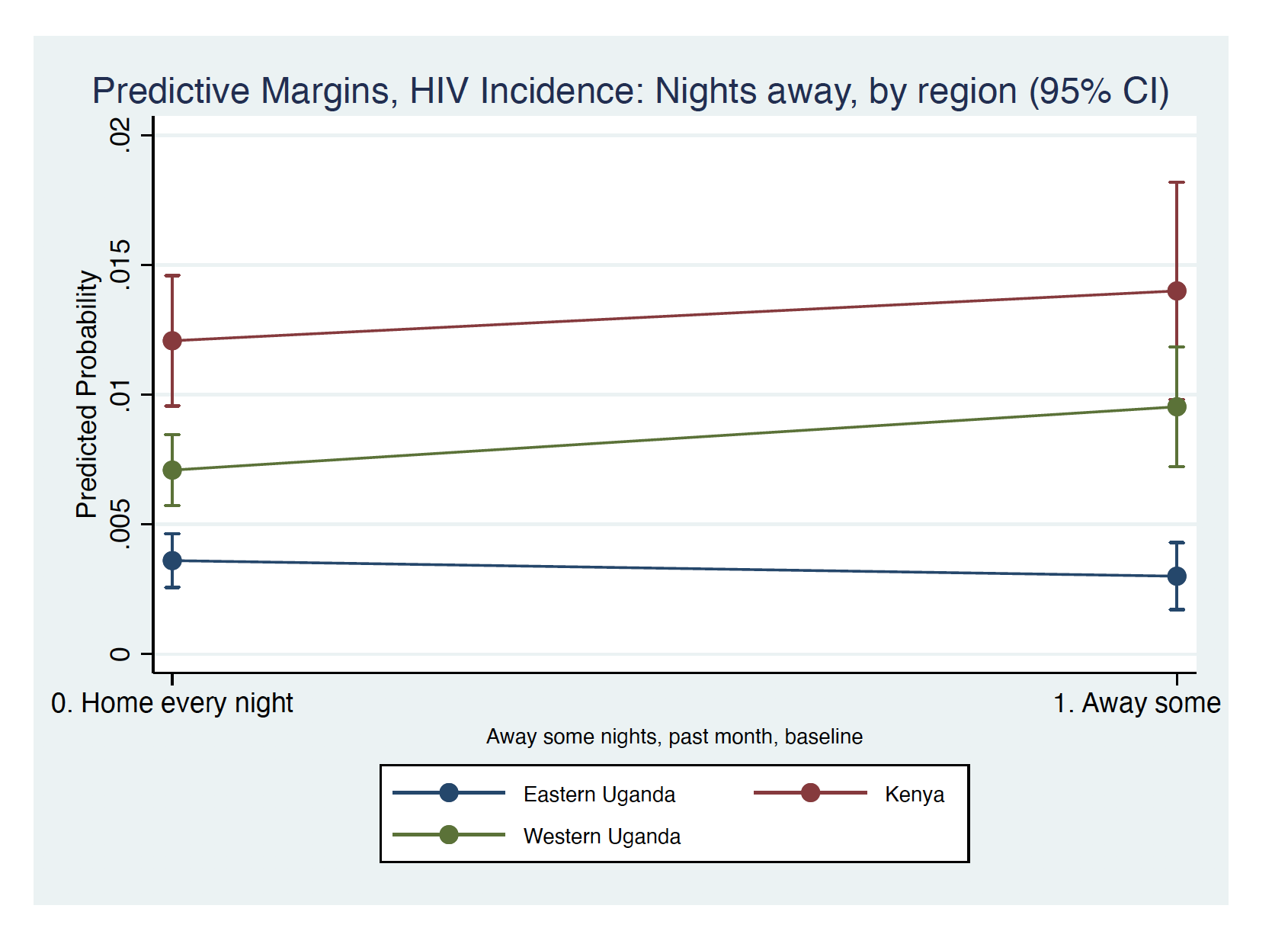
**

Notes: Margin plots show predicted probability of HIV acquisition over the three year period by region, from multivariable Poisson regression models to examine moderating effect of region on effects of mobility metrics on HIV acquisition, i.e. hypothesis that the relationship between a) Having spending at least some nights away from household at baseline and b) Having spent >=1 month away from household at year 3, and risk of HIV acquisition, is moderated by region.
